# The Impact of Mandate Contract and Self-Employment on Workers’ Health—Evidence from Poland

**DOI:** 10.3390/ijerph18063138

**Published:** 2021-03-18

**Authors:** Katarzyna Piwowar-Sulej, Dominika Bąk-Grabowska

**Affiliations:** 1 Department of Labor Capital and Innovation, Faculty of Management, Wroclaw University of Economics and Business, Komandorska St. 118/120, 53-345 Wrocław, Poland; 2 Department of Economics and Organization of Enterprise, Faculty of Management, Wroclaw University of Economics and Business, Komandorska St. 118/120, 53-345 Wrocław, Poland; dominika.bak-grabowska@ue.wroc.pl

**Keywords:** non-standard employment, flexible employment, physical health, mental health

## Abstract

The purpose of the study is to analyze the correlations between two clearly defined forms of non-standard employment (self-employment and mandate contract) and workers’ health. The study also addressed such variables as gender, age, length of service, and the reason for employment (voluntary vs. non-voluntary). The research was carried out in Poland in 2020 using the CATI method (a telephone interviewing technique), and it covered a sample of 200 workers (100 self-employed and 100 working under a mandate contract). Most of the respondents declared that their form of employment did not affect their health. However, the statistical analysis showed significant differences in health status between the self-employed and those working on a mandate contract. Self-employed respondents experienced mental health impacts more often, whereas those working under a mandate contract more frequently declared that their physical health was affected. The length of service was only important for mental health, having a negative impact on it. The respondents’ age and gender turned out to be statistically insignificant, which is in contradiction to many previous research findings. The inability to choose one’s form of employment resulted in worse physical health. These findings demonstrate the importance of certain variables that were not prioritized in previous studies and emphasize the need to clearly define what non-standard and precarious forms of employment are, as well as revealing new correlations between the studied categories and providing directions for further research.

## 1. Introduction

The standard, traditional form of employment is secured with an open-ended, full-time—and thus stable—employment contract. Nowadays, new forms of employment are emerging that differ from the traditional employment contract [1]. Non-standard forms represent an increasing proportion of employment in much of Western Europe. As reported in 2016 by the European Parliament, the frequency of standard employment contracts declined from 62% in 2003 to 59% in 2014 [2].

The shift toward a more flexible labor market have focused the attention of researchers on the health effects of downsizing, temporary employment, and job insecurity [3]. Many review studies have confirmed the correlation between precarious employment and several dimensions of health. At the same time, it is recommended to continue research projects focused on expanding and clarifying the selected issues [4], because many previous studies designed to determine the impact of precarious forms of employment in fact used a category of non-standard employment [5]. For example, specific recommendations include clearer, more precise definitions of the original concepts that take into account the fact that non-standard forms of employment and precarious employment are defined differently, a more detailed understanding of the pathways and mechanisms through which non-standard employment harms workers’ health, stronger information systems and a complex systemic approach to employment conditions, and identification of which groups of employed people bear the highest risk [4,6]. Moreover, a shortage of research based on primary, high-quality data and analysis, aimed at confirming the correlation between employment and worker health [5], has been indicated, whereas the existing studies have not solved the outlined problems (the research remains in its infancy) [4].

The negative impact of non-standard employment on worker health is manifested by such factors as job insecurity [7,8,9] or perceived stress [10,11]. The assumption that these factors characterize all possible non-standard forms of employment may not always be true, due to the numerous and different types of non-standard employment found in different countries. For example, in Great Britain, a specific type of contract is used between an employer and an employee: a zero-hour contract, which provides almost complete flexibility in arranging the employee’s working hours, with the worker retaining the status of an employee [12]. In Poland, in turn, it is possible to conclude a contract with a worker, who is a natural person, based on civil law rather than labor law, such as a mandate contract or a contract to perform a specified task. In such cases, the worker does not hold the status of an employee.

The aforementioned various forms of employment may have a different effect on workers’ health. Therefore, it is justified to directly analyze the impact of individual forms of employment on self-reported health as well as the ailments reported by workers. In addition, the results of the existing research in most of the analyzed areas are not clear-cut (e.g., in relation to gender as a variable that influences health (cf. [13,14]), which also indicates the need for further exploration.

The unresolved problems (research gaps) can be defined as follows:

1. The absence of a clear distinction between precarious and non-standard employment may result in extending the conclusions drawn from the research on precarious employment to the category of non-standard forms of employment. Therefore, research addressing precisely defined forms of non-standard employment is needed.
2. The category of non-standard forms of employment is not a homogeneous one, since particular forms of non-standard employment have varying characteristics. Therefore, apart from synthesizing the research results, it is also necessary to separately consider the impact of certain forms, such as self-employment, on workers’ health.
3. When determining the impact of forms of employment on workers’ health, such variables as gender or age are emphasized; however, the results depicting the actual impact of these variables are not clear-cut.
4. Given that the categories of precarious and non-standard employment are not identical, a new approach is needed to define the variables in research addressing the impact of non-standard employment on workers’ health.
5. There is a need for research not only based on an analysis of secondary data (from the available macroeconomic databases) but also targeting workers employed in non-standard forms and designed to identify the impact of these non-standard forms of employment on their health.

The above problems have become the basis for research to assess the impact of employment based on the two most popular non-standard forms of employment in Poland on health, taking into account the employees’ perspective. Poland is an interesting geographic area for research addressing non-standard forms of employment due to the fact that this country significantly exceeds the average for EU countries in terms of the percentage of both employees with temporary contracts (fixed-term employment and civil-law contracts) and precarious workers [15,16].

The purpose of the article—in its theoretical part—is to determine the health consequences for workers of non-standard employment and precarious employment, as well as to investigate the factors taken into account in the research conducted so far on the impact of non-standard and precarious forms of employment on workers’ health. Moreover, a conceptual and taxonomic goal is to categorize what is meant by non-standard forms of employment and when employment becomes precarious. The research methods used to achieve these theoretical and conceptual goals took the form of literature studies (including, for example, the research findings presented in articles indexed in the Web of Science and Scopus databases) and desk research based on data from public statistics.

The compilation and analysis of information from secondary sources led to the formulation of five research hypotheses, whose verification became the empirical goal of the article. These hypotheses refer to correlations between (1) non-standard forms of employment and their impact on mental and physical health, (2) length of service and the impact of non-standard employment on mental and physical health, (3) the respondent’s gender and the perceived impact of non-standard employment on mental and physical health, (4) the respondent’s age and the perceived impact of non-standard employment on mental and physical health, and (5) the reason for employment (voluntary vs. non-voluntary) and the perceived impact of non-standard employment on mental and physical health.

Physical health can be defined as normal functioning of the body at all levels; a normal course of biological processes that ensures individual survival and reproduction; a dynamic balance between the body’s functions and the environment; participation in social activities and socially useful work; performance of basic social functions; the absence of diseases, painful conditions, and changes; and the body’s ability to adjust to the constantly changing conditions of the external environment, [17] (p. 603), whereas mental health can be defined as “a state of well-being in which the individual realizes his or her own abilities and is able to cope with the normal stresses of life, work productively and fruitfully, and make a contribution to his or her community” [18].

The structure of the article corresponds to the above-presented objectives. The section following the Introduction presents the literature background and hypotheses. Then, the methodology of the empirical research is described. The following part of the article presents the results and discusses the findings. The last section includes not only conclusions but also describes the study’s limitations and suggests possibilities for further research.

The article contributes to the development of the field of knowledge—non-standard forms of employment and their implications for workers—through (1) an analysis of the existing research covering the impact of non-standard and precarious forms of employment on workers’ health (including an analysis of the effects of using specific forms of employment and additional factors—apart from the form of employment—taken into account in previous research projects), (2) categorizing such concepts as non-standard employment and precarious employment, (3) presenting the results of an original research project focused on two specific forms of non-standard employment and two dimensions of health (physical and mental health), and (4) identifying directions for further empirical research.

## 2. Literature Background and Hypotheses

### 2.1. Non-Standard and Precarious Employment vs. Workers’ Health

As indicated in the Introduction, many review studies point out that the correlation between precarious employment and several dimensions of health has been confirmed [4]. A study for Korea using longitudinal data reported a significant health-damaging influence from flexible employment [19]. For example, worsened subjective assessments of health were previously observed among people employed under non-standard forms in Sweden [20] and in the Netherlands [21].

Some research has shown the impact of non-standard employment on experiencing physical ailments. Several studies have proven that there is a correlation between temporary employment and musculoskeletal disorders. Silvestrone et al. [22] revealed that temporary workers in the USA complained of back and neck pain more often than regular employees. Similar results were obtained in a group of Swedish workers [20]. In turn, lower back pain was observed much more often among fixed-term employees than permanent employees in Spain [23]. Research conducted in Japan also confirmed the correlation between non-standard employment and the risk factors responsible for cardiovascular diseases [24].

Previous research has also addressed the link between workers’ health and the feeling of insecurity and non-standard working hours based on the example of home care workers [7]. In that study, workers’ health was defined in terms of stress and musculoskeletal disorders, non-standard working hours were identified as part-time casual work, and uncertainty was related to work and job insecurity. Quantitative research covering a group of 532 home care workers (nurses, physical therapists, and personal support workers) showed that non-standard working hours and job insecurity are strongly associated with a feeling of stress. The relationship between non-standard working hours and musculoskeletal disorders is moderated by the symptoms of worker stress, and the correlation between employment insecurity and musculoskeletal disorders does occur and is direct. Referring to these results, it is possible to point out the significant importance of stress as a variable that leads to deterioration in health. Benavides [11] studied cross-sectional data from 15 European countries and reported that “precarious” employment was more stressful.

Some studies have analyzed correlations between non-standard employment and poorer health in relation to various forms of non-standard employment, and in some cases, this correlation is shown only in relation to selected forms of employment. Artazcoz et al. [25] found that employment through a temporary employment agency alone was associated with worse health indicators and, at the same time, showed that the result was dependent on the gender and social class of the temporary workers. Using longitudinal data from Britain and Germany, Rodriguez [26] found that only fixed-term employees in Germany reported significantly lower mental health. Therefore, it is worth pointing out that research that takes into account specific forms of non-standard employment is valuable. The above considerations led to the formulation of the first hypothesis.

**H1.**  *There is a statistically significant difference regarding the perceived impact of the form of employment on health between workers employed in different non-standard forms of employment*.

An important research area discusses correlations between job insecurity related to non-standard employment and workers’ mental health. One of the systematic reviews covering research on fixed-term employment, job insecurity and its impact on mental health showed a strong correlation between job insecurity and the incidence of mental illness [27]. The outcomes of this review suggest that the engagement in fixed-term employment and the related job insecurity determine workers’ health to a significant extent. It was also found that the consequences for health of fixed-term employment may be mitigated by certain attitudes presented by employees in relation to the form of their employment, which is correlated with, for example, the voluntary choice of a given form of employment.

A study conducted in the United States [8] showed the influence of persistent job insecurity on workers’ anxiety and their mental condition. It was found that persistent job insecurity was strongly and significantly associated with greater psychological distress, primarily among older workers. Dockery [28] found that both working non-standard hours and job insecurity reduced mental health for Australian workers. Aronsson and Goransson [20] observed that temporary employment was connected with higher levels of depression and fatigue in Swedish workers. Virtanen et al. [29] demonstrated that Finnish temporary workers were characterized by an increased level of hostility, aggravation, and depression. The correlation between precarious employment and the symptoms of depression and dysthymia was confirmed by studies conducted in Italy [30]. Another study showed that part-time work experience had a direct correlation with poor mental health and suicidal behavior [31]. In turn, Vives et al. [32], who analyzed Spanish workers employed on temporary contracts, confirmed that people with short-term employment contracts were more likely to suffer from depression than those in permanent employment. Benavides [11], while analyzing the results of the European Work Conditions Survey, additionally stated that fixed-term employment is significantly and positively related to worker fatigue. In turn, self-employment is associated with sleeping disorders [33].

However, in this area, it is also possible to mention research findings that do not confirm the negative impact of jobs based on precarious and/or non-standard employment on mental health. A study carried out in Australia found that in most cases, there was no direct relationship between mental health and either the current or a previous form of employment. For the current period of employment, the exception was a higher level of mental health for women employed as full-time casual workers. The only support for the proposition that flexible employment harms mental health was in the case of men employed under fixed-term, full-time contracts [34]. Another study carried out in Great Britain did not confirm any significant health consequences of precarious employment [35], whereas a study covering employees with fixed-term employment even showed that they have better self-rated health than their permanent counterparts [36].

The research presenting the multidimensional impact of precarious employment on workers’ health can be considered pioneering. One such project investigated correlations between general health, mental health, and musculoskeletal pain. The authors concluded that their findings strengthened the existing evidence on the harmful impacts of precarious employment, while pointing to the need for further research in the area of both physical and mental health [37]. This became an important premise for the formulation of the research sub-hypotheses within the framework of the first hypothesis.

**H1a.**  *There is a statistically significant difference regarding the perceived impact of the form of employment on physical health between workers employed in different non-standard forms of employment*.

**H1b.**  *There is a statistically significant difference regarding the perceived impact of the form of employment on mental health between workers employed in different non-standard forms of employment*.

### 2.2. Variables Used in the Research on the Impact of Non-Standard and Precarious Forms of Employment on Workers’ Health

Evidence confirming the negative impact of precarious employment and non-standard forms of employment can be found in the research carried out in various countries. Apart from analyzing the general influence of the form of employment on different types of health (physical vs. mental), authors have introduced numerous detailed variables, such as job security/employment stability, industry (position), and the age, gender, and (dis)ability of the respondent.

When presenting the variables, primarily the problem of job insecurity was addressed, presenting results that mainly showed a positive health impact of stable forms of employment and a negative one of unstable forms related to job insecurity. It is worth clarifying that in the existing studies, instability and job insecurity were most often a priori attributed to non-standard forms of employment. However, the concept of this study approaches instability as a variable. It was decided that the non-standard forms of employment do not have to be associated in every single case with significantly less stability and a significantly lower sense of security. Therefore, when formulating the second research hypothesis, reference was made not to the category of job stability or security but to the actual length of service in a given organization as part of work in a specific form of employment.

Virtanen et al. [38] conducted research among Finnish employees working in municipal offices. They found that permanent employees self-reported their health as better. They were also diagnosed with fewer chronic diseases than in the case of those employed based on temporary employment contracts. A study conducted among Belgian doctors also confirmed that those working on fixed-term contracts subjectively assessed their health as worse and experienced distress more often than the doctors employed based on permanent contracts [39]. Research addressing the importance of unstable employment for workers’ health in Great Britain was also carried out among people of working age with and without intellectual disability [9]. The analysis of correlations between employment conditions and self-reported general health among British adults showed that people with intellectual disabilities were exposed to unstable employment conditions and experienced job insecurity more often than their peers. The findings covering both the disabled and the healthy respondents showed that the experience of insecure employment is correlated with worse health status.

The experience of insecurity among workers is an important mediating variable taken into account when examining the impact of the type of employment on health. There is a growing body of international evidence that experiences of insecurity as well as high demands and powerlessness in employment have health consequences, even though the findings are not yet conclusive [38,40]. The significant increase in using non-standard forms of employment along with a high unemployment rate and professional inactivity rates were the premises for research conducted in Italy to determine whether job insecurity and a lack of job opportunities affect physical and mental well-being differently than standard employment under an open-ended employment contract [41]. The analysis of data from Italy, from the Survey on Household Income and Wealth, showed that self-reported health status was related to employment status (including the form of employment). Temporary workers, first-time jobseekers, and the unemployed were worse off than permanent workers. According to the authors, this applied primarily to men and young workers. Most of the published research documented adverse effects on health. Job insecurity has been identified as a major pathway linking non-standard forms of employment with negative health outcomes, and meta-analyses have confirmed the significant associations between them [42,43,44,45]. This correlation is also confirmed by the latest research: the perceptions of insecurity in current employment and the dimension of precarious employment are strongly associated with poorer self-reported health [46].

A similar category, used in some studies as a mediating variable between the form of employment and health status, is “employment stability”. Interesting findings resulted from a study conducted in Spain that examined the relationship between job stability and mental health, taking into account the criteria of gender and partner/marital status. A total of 6859 men and 5106 women, both working and unemployed, were analyzed based on the data from the National Health Survey. The measure of employment stability was introduced, and mental health was determined using 12 points from the General Health Questionnaire. The results showed that in all groups except married women, the factor of job stability was related to mental health. Among married and cohabiting couples, this correlation was stronger in men. The strongest correlation was observed among either separated or divorce individuals [47].

However, it is worth noting that subjective job insecurity/employment stability is only one dimension of non-standard forms of employment and that focusing solely on this dimension does not provide a comprehensive picture of the impact of these forms of work on workers’ health status [34,48]. Moreover, even when focusing on the “job insecurity” category, the results are inconclusive. Virtanen et al. [49] found that exposure to temporary employment did not add to the harm done by feelings of job insecurity.

One possibility to determine the importance of experiencing job insecurity in the impact of non-standard forms of employment on workers’ health may be introducing the category of length of service into research. A worker employed in a non-standard form of employment at a given company for a relatively longer period of time should experience job insecurity to a lesser extent, which should be related to their perceived health condition. So far—according to the conducted review—such a correlation has not been defined as the subject of research. It is presented in the second research hypothesis.

**H2.**  *There is a statistically significant difference in the perceived impact of different forms of employment on workers’ (physical (H2a) and mental (H2b)) health, depending on the length of service in the current form of employment*.

Gender is one of the significant factors taken into account when conducting research on the impact of non-standard employment on workers’ health. It has been indicated that employment conditions tend to be gendered, with women carrying the largest burden of precarious employment. In addition, there is a clear gender difference under some forms of employment: women are much more likely than men to be employed under casual terms and part-time [34]. Even though some studies suggest that precarious employment may have a greater impact on women’s health than in the case of men, it is believed that our knowledge in this area is limited [50]. Moreover, working women are identified as more exposed to negative health effects, regardless of the form of employment, and certain factors, such as having school-aged children or no autonomy in the workplace, may even intensify these negative effects [51].

An example of a study that confirmed the greater harmfulness of non-standard employment to women’s mental health is an analysis that showed a significant correlation between fixed-term employment and depression only in the group of women [52]. Similar findings were demonstrated in South Korea—symptoms of depression were observed only in women with fixed-term employment [13]. On the other hand, German researchers who measured the tendency for self-rated health to coexist with various dimensions of precarious employment, taking into account the gender criterion, found a greater impact of job insecurity on men’s health [14]. It is believed that the gender factor should be considered when analyzing the impact of non-standard forms of employment on workers’ health, also in connection with other variables, thus developing knowledge about particularly vulnerable groups [4]. It provides grounds for conducting further research in this area that would include gender as a criterion, which was expressed in the next research hypothesis.

**H3.**  *There is a statistically significant difference in the perceived impact of different forms of employment on workers’ (physical (H3a) and mental (H3b)) health, depending on the respondent’s gender*.

It is argued that apart from women, groups that are particularly vulnerable to precarious employment are youths and older workers, as well as less-educated people and migrants [53]. It is worth analyzing correlations not only between such factors as age and the frequency of work under non-standard forms but also the influence of age on the perceived health of workers employed under these forms, as emphasized in the following hypothesis.

**H4.**  *There is a statistically significant difference in the perceived impact of different forms of employment on workers’ (physical (H4a) and mental (H4b)) health, depending on the respondent’s age*.

As mentioned in the introduction, authors have postulated continuing research projects focused on deepening and clarifying the selected issues [4]. Voluntary work in non-standard forms, which affects workers’ attitudes, may be one of such important variables. It is suggested that positive attitudes presented by workers may partially eliminate health consequences—and positive attitudes (a good frame of mind) are reinforced by the possibility of choosing the form of employment [27]. Therefore, in the conducted analyses, it is worth considering the reasons for which workers take up non-standard forms of employment and examining the impact of this factor on their perceived health status. At this point, it is worth noting that self-employed people decide for themselves the level of costs generated by their activity and therefore have an impact on the amount of income tax they pay. They can also choose different forms of taxation, whereas full-time employees (based on the Labor Code) are taxed in line with the progressive tax scale [54]. In turn, B2B (business to business) contractors (self-employed people) can benefit by paying lower social security contributions. They decide whether they want their sick leave to be covered by insurance, and even if they do, their contributions are capped at a certain threshold [55]. These considerations became the premise for formulating the final research hypothesis.

**H5.**  *There is a statistically significant difference in the perceived impact of different forms of employment on workers’ (physical (H5a) and mental (H5b)) health, depending on the reason for taking up non-standard employment*.

It is worth mentioning that some publications have linked the problem of the impact of precarious employment on health with the specific working conditions of a given industry or profession, e.g., the sex industry [56]. Even if the very nature of the industry’s working conditions seems to have an adverse effect on health, the results of some studies are inconclusive. For example, a study conducted in Great Britain regarding women working in the stripping industry (“lap dancing”) found that women who decide to perform this job—classified as a precarious form of employment—often thought about it in strategic terms. They were likely to approach it as a way to achieve security for the future, investing in their education, and finding new employment in the future, which translated into a better impact on their health [57]. Thus, the factor of time is revealed here. Working in precarious forms of employment may allow a worker to collect the capital needed to find safer and more prestigious employment.

### 2.3. Non-Standard Forms of Employment and Precarious Employment—Toward Identifying a Precise Semantic Approach and Determining the Scope of Application

As mentioned in Section 2.1 and Section 2.2, in the studies addressing the impact of forms of employment on workers’ health, researchers relatively often refer to the category of precarious employment. Despite the growing use of the term “precarious employment”, there is no consensus on a theoretical framework or definition [58]. The term is often applied to jobs that are casual, contract, labor-hire, or not full-time; it implies a disadvantage [34]. Standing’s “precariat”—described as a “class-in-the-making”—is characterized by chronic uncertainty and insecurity, and though it is still divided within itself, it represents an alternative approach to precariousness because it focuses on the capacity of workers in precarious jobs to act collectively in their own interest (i.e., as a class). While defining the precariat, Standing clearly indicated the importance of age and gender [59].

Taking another perspective, the precariat refers to employment as a multidimensional construct that encompasses dimensions of employment insecurity, individualized bargaining relations between workers and employers, low wages and economic deprivation, limited workplace rights and social protection, and powerlessness to exercise legally granted workplace rights [60]. Moreover, it has been indicated that when analyzing precarious employment, researchers should refer to the conditions of national job markets [4]. This is an important remark, as the social systems and legal norms that determine the possibility of using certain forms of employment, for example, differ from country to country.

The term “precarious employment” appeared in the debate, initially mainly within Europe, in close connection with the increase in non-standard forms of employment [34]. Analysts were concerned that workers were increasingly exposed to job insecurity and denied many of the benefits that came with standard employment, such as paid leave, unemployment insurance, health benefits, and training [61]. In systematic reviews addressing the correlations between precarious employment and workers’ health, precariousness is shown in the search strategy through various terms: outsourced, outsourcing, temporary, atypical, contingent, atypical, flexible, casual, non-standard, or nonstandard; these are combined with such terms as employment, work, and job [5]. This results in combinations (definitions of forms of employment) not all of which—as it seems—are necessarily of a precarious nature. This may partly explain the discrepancies in the research findings.

**Figure 1:**
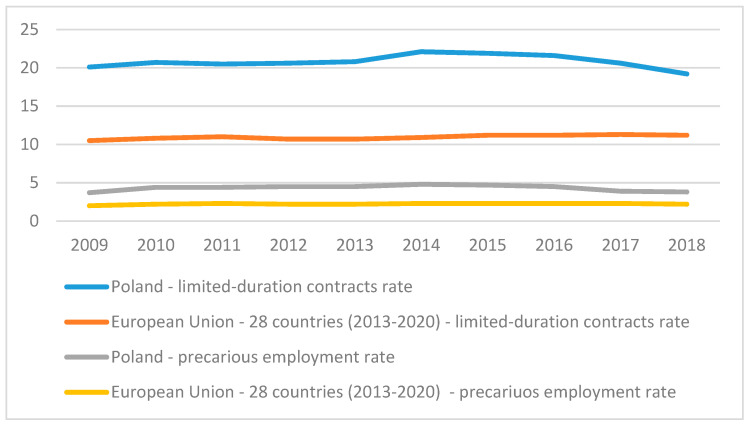
Poland at the background of the EU average in terms of non-standard forms of employment based on [14,15].

The research conducted in subsequent periods confirms an increase in the use of non-standard forms of employment. The proportion of individuals employed on flexible terms, including part-time, casual, fixed-term contracts, labor-hire, and self-employment, has increased greatly in all industrial countries since the 1980s [62]. The increase in using non-standard forms of employment and the related higher job insecurity was also demonstrated in the first decade of this century, in particular in English-speaking countries and the countries of southern and eastern Europe [63]. The current data also confirm the significant proportion of non-standard and precarious employment, with Poland standing out from among the countries of the European Union (Figure 1).

The dissemination of non-standard forms of employment has led to research efforts being concentrated on determining the impact of such work on workers’ health. This need is strongly emphasized from the perspective of public health but also from the standpoint of psycho-social working conditions and human resource management, including the assumptions related to the concept of sustainable human resource management [4,45,64,65]. Due to the extensive application of non-standard forms of employment in Poland, and also the occurrence of specific forms, based on civil law rather than labor law, the need for conducting such research in Poland becomes particularly evident. As Table 1 shows, in Poland self-employment is the most frequently used non-standard form of employment. Mandate contract is the second most common type.

In Poland, the term self-employment typically refers to individuals, but the legal status of self-employed workers may differ depending on the country. Self-employment is not a homogeneous category; it is a complex and ambiguous phenomenon that presents problems in defining it [66]. Boegenhold [67] pointed out that the majority of studies try to relate to the enigmatic typical self-employment, which does not exist in practice. When examining macro-level patterns of self-employment, a number of patterns emerge. The author demonstrated that self-employment covers both marginal and privileged positions in individual countries as well as internationally. It can put people at risk of precariousness and poverty, or it can serve as a tool of generating wealth for individuals and companies, contributing to job creation and economic growth for society. In addition, hybrid forms of employment are increasingly common, in which people combine self-employment with traditional employment [67]. Self-employment, to a larger extent, seems to be treated as a form of employment that offers the opportunity to work independently, develop one’s career, practice entrepreneurship, and earn more than through traditional employment [68]. Therefore, when examining the impact of non-standard employment on workers’ health, it is worth approaching self-employment as a separate category.

Not all countries use the form of employment based on mandate contract. In the Polish legal system—according to the case law of the Supreme Court—employment does not have to be employee-related and may result from civil-law contracts. It is accompanied by a basic division of employment arrangements into employee-related and non-employee-related. The latter type involves working under one of the civil-law contracts. This leads to a specific work relationship that positions the contractors outside the binding labor law system, and in particular cases outside the system of obligatory social security [69]. It seems that work based on a civil-law contract (e.g., mandate contract) may demonstrate the characteristics of precarious employment to a greater extent. This becomes an argument for separately considering the impact of these forms on the well-being and health status of workers, followed by comparisons.

The above discussion illustrates that the term “non-standard forms of employment” also remains ambiguous. On the one hand, these forms can be defined relatively broadly and generally as the forms that differed from the “masculine” norm of full-time, permanent, year-round employment [34]. However, on the other hand, they can be defined in narrower, more precise terms, at the same time pointing to various forms of non-standard employment.

In this research project, a narrower understanding of non-standard forms of employment was adopted, which was based on the criterion of the type of contract/agreement between the employer and the worker. If it is an employment contract based on the labor law of a given country, signed directly with the worker, it is treated as the standard form. Non-standard forms cover self-employment, civil-law-based contracts, such as a mandate contract or a contract to perform a specific task (used in selected countries), agency employment, and or some types of outsourcing (agency or outsourced workers) [70].

**Figure 2:**
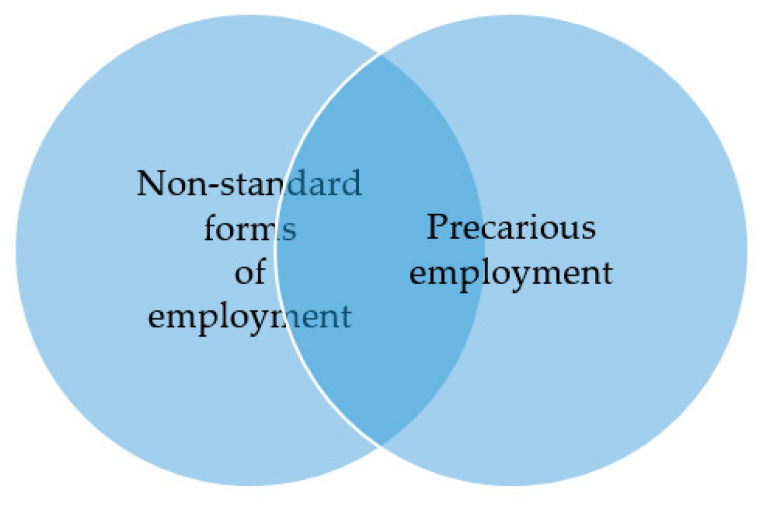
The correlation between non-standard forms of employment and precarious employment.

Due to the prevalence of self-employment and mandate contracts in Poland, the assumptions of the research project focused on these two forms of non-standard employment. At the same time, it was not assumed a priori that these forms were synonymous with precarious work. If a narrower understanding of non-standard forms of employment is adopted, then, on the one hand, not every single precarious employment relationship is necessarily a non-standard one, and on the other hand, not every form of non-standard employment is necessarily precarious by nature (Figure 2).

Full-time employment under a traditional working time organization system may present some characteristics of precarious employment, such as a very low salary. In turn, work in non-standard forms such as self-employment may be associated with good working conditions, high income, and meeting workers’ expectations—hence the postulate of examining the individual non-standard forms of employment separately, which may provide in-depth knowledge about the nature of work based on these forms [10].

## 3. Material and Methods

The empirical research focused on answering the following research question: How do people employed in non-standard forms of employment (without any additional standard employment) assess the impact of this employment on their health? As indicated above, the focus was on such forms of non-standard employment as mandate contract and self-employment, due to the prevalence of these forms in Poland.

In addition, the following research hypotheses were formulated:

**H1.**  *There is a statistically significant difference regarding the perceived impact of the form of employment on workers’ (physical (H1a) and mental (H1b)) health between those employed in different non-standard forms of employment (in this case, mandate contract and self-employment).*

**H2.**  *There is a statistically significant difference in the perceived impact of different forms of employment on workers’ (physical (H2a) and mental (H2b)) health, depending on the length of service in the current form of employment*.

**H3.**  *There is a statistically significant difference in the perceived impact of different forms of employment on workers’ (physical (H3a) and mental (H3b)) health, depending on the respondent’s gender*.

**H4.**  *There is a statistically significant difference in the perceived impact of different forms of employment on workers’ (physical (H4a) and mental (H4b)) health, depending on the respondent’s age*.

**H5.**  *There is a statistically significant difference in the perceived impact of different forms of employment on workers’ (physical (H5a) and mental (H5b)) health, depending on the reason for taking up non-standard employment*.

Due to the similar numbers of people in the general population employed on a mandate contract and the self-employed (Table 2), it was decided to include in the study 100 people representing each form of employment. An important criterion for the selection of respondents—apart from the form of employment (approached as permanent rather than casual employment)—was a minimum length of service under the current form of employment of one year. The authors decided after consulting the topic with a psychologist that one year is a sufficient period to observe the impact of the form of employment on a worker’s health.

A pilot survey covering a sample of 30 respondents was conducted in June 2020. It allowed the researchers to verify the original research instrument (questionnaire). A proper survey using the telephone interviewing technique (CATI) was conducted between July and September 2020. A professional research agency was involved in collecting the data. A random selection was done among companies that must report official statistics on their use of non-standard employment. Then, the survey was conducted on workers, with their and their employers’ approval. The research was anonymous. Table 2 presents more detailed characteristics of the research sample.

Table 2 presents only the data that were used in further statistical analysis, but during the research, more detailed information about the study sample was collected. This allows for a more precise understanding of the workers’ situation and is therefore briefly presented below.

The trade and service industries were most heavily represented in the survey (43 respondents each, which amounts to 21.5% of all respondents). Moreover, after a more detailed analysis of the demographics, it should be stated that self-employed people (apart from the indicated industries, wherein 18 people work in trade and 31 in services) primarily represented the medical (14 people) and manufacturing (11 people) industries. In turn, the people employed on mandate contracts (apart from the trade and service industries, which applied to 25 and 12 people, respectively) mainly worked in the construction and hospitality industries (9 people each). Eighty (40% of all respondents) had a secondary-school education, and the second-most represented group (58 people, 29% of all respondents) was workers with a master’s (five-year) degree.

The question about the notice period also yielded interesting information. According to the Polish Labor Code, the basic notice period is either two weeks (when the employment contract has been valid for a period shorter than six months), one month (when the employment has lasted between six months and three years), or three months (when the employment has lasted at least three years) [71]. As shown in Table 2, in the case of persons employed based on non-standard forms, longer notice periods are applied, which should increase their sense of job security.

As far as the reliability of the scale is concerned, it is worth noting that the questions included in the research instrument directly pertained to the variables under study. The questions referred to information about the variable (variable definition). The researchers were interested in the type of impact (negative or positive), not the extent of this impact, and the respondent’s subjective assessment of the impact. When asked about the perceived impact of the form of employment on physical and mental health, the respondents were given an appropriate definition of the type of health and were asked to choose one of the following answers:

(a) No, it has no impact,
(b) Yes, it has an impact—working in this form resulted in better health for me,
(c) Yes, it has an impact—working in this form resulted in worse health for me,
(d) I don’t know/haven’t thought about it.

For the purposes of statistical analyses, the answers “I don’t know” and extreme answers (concerning the positive impact of the form of employment on the respondent’s health) were excluded.

## 4. Results and Discussion

Even though the vast majority of respondents admitted that their form of employment did not affect their health, the analysis showed a statistically significant correlation between the form of employment and the perceived impact of this form on both physical health (χ2[1, N = 194] = 10.171; *p* = 0.001) and mental health (χ2[1, N = 184] = 6.153; *p* = 0.013). In the former case, the people employed on a mandate contract more often experienced negative impacts on their physical health from their form of employment. In the latter case, it is the self-employed that more often notice the negative impact of their employment on their mental health (Table 3). Thus, the first hypothesis—which assumes there are differences between these forms of employment in the context of affecting workers’ health—and its sub-hypotheses (for each type of health) were confirmed.

In an open-ended question, 39 respondents indicated examples where impacts to health were the consequence of the form of their employment. So, with regard to physical health, the most frequent complaints were back pain (*n* = 16), muscle pain (*n* = 6), joint pain (*n* = 5), and leg pain (*n* = 3). The respondents emphasized that back pain is related to both lifting heavy objects or people (e.g., in the medical industry) and sedentary work. When it comes to mental health, 26 respondents declared symptoms of deteriorated health. They listed the following symptoms most frequently: stress (*n* = 14), feeling blue (*n* = 3), nervous tension (*n* = 2), short temper (*n* = 1), and depression (*n* = 1). They associated depression with the longest and the most extreme case (inability to function normally). Stress—according to the respondents—resulted from time pressure, a lack or excess of work (tasks to be performed), work control, customers’ behavior, delays in receiving payment for their work, and emotional involvement in work.

The analysis did not show a statistically significant correlation between the length of service and the perceived impact of the form of employment on physical health (χ2[3, N = 194] = 3.350; *p* = 0.341). However, the length of employment period was significant for the perceived impact of the form of employment on the respondent’s mental health (χ2[2, N = 184] = 18.430; *p* < 0.001). The negative impact was noticed three times more often by people working for more than 5 years under the same form of employment (Table 4). Thus, the second hypothesis was only partially confirmed—the assumptions presented in sub-hypothesis H2b. This means that there was a statistically significant difference between forms of employment in the length of service in terms of the perceived impact on workers’ mental health.

Neither the respondent’s gender nor age was significant in the context of their perception of the form of employment having an impact on their health. The following results were obtained for physical and mental health, respectively, for the gender variable: (χ2[1, N = 194] = 2.836; *p* = 0.092) and (χ2[1, N = 184] = 0.370; *p* = 0.543); for the age variable, it was (χ2[3, N = 194] = 3.350; *p* = 0.341) and (χ2[3, N = 184] = 2.024; *p* = 0.567), respectively. Thus, the third and the fourth hypotheses regarding the relationship between gender and age, respectively, and impact of the form of employment on workers’ health were not confirmed.

The distribution of responses about the perceived impact from the form of employment on physical health in relation to the reason for taking up employment is presented in Table 5.

In this case, the analysis showed a statistically significant correlation between the variables (χ2[2, N = 194] = 14.406; *p* = 0.001). The perceived negative impact from the form of employment on physical health was noticed three times more often by people who did not have the option to choose their form of employment than in the case of those who were guided by financial considerations. On the other hand, no such relationship was identified between the reason of employment and the perceived impact of the form of employment on the respondent’s mental health (χ2[2, N = 184] = 3.239; *p* = 0.198). Thus, the fifth hypothesis was only partially confirmed in relation to the assumption presented in sub-hypothesis H5a. This means that there is a statistically significant difference between the forms of employment in the respondents’ perception of its impact on their physical health in terms of their reason for taking up non-standard employment.

The research shows that for the vast majority of respondents, the form of employment has not affected their health. Therefore, these findings are in contradiction to those of studies by Aronsson and Göransson [20], Klein Hesselink et al. [21], Kawachi [40], and Kim [19]. At the same time, as shown above, there is a statistically significant correlation between such variables as the type of employment (mandate contract vs. self-employment) and the perceived impact of this type of employment on health (physical and mental). So far, researchers have generally focused on people employed on limited-duration contracts or agency workers and have not compared the types of non-standard employment; therefore, there is no reference in the literature to the regularity detected here.

Contractors more often cited the negative impact of this form of employment on their physical health, while self-employed people more frequently reported a negative impact on their mental health. It seems that the reason for this situation may lie in the nature of the work, which, in turn, is related to the industry in which a given respondent works. Although the characteristics of the research sample made it impossible to carry out a cross-industry statistical analysis, as the demographics show, the respondents from both groups worked to a large extent in similar industries.

The symptoms of worse physical health are consistent with previous findings [20,22,23]. As far as mental health is concerned, stress was reported by the respondents as the most common manifestation of deteriorated health. Here, stress should be approached more as a cause rather than an effect. Stress—according to the respondents’ opinions—results primarily from time pressure or having too many or no tasks to perform. Therefore, it is not closely related to the form of employment, but rather to the specific job. None of the respondents indicated—as was reported by Dockery [28], Zeytinoglu et al. [7], and Burgard et al. [8]—that their employment is accompanied by non-standard working hours and job insecurity which, in turn, would be causes of stress.

In one case, the precariousness of the form of employment may be demonstrated as a source of stress, i.e., delays in receiving payment for work performed. The law is not as restrictive in this matter as in the case of employment contracts, and the process of extending such rights to self-employed people or contractors is longer and more complicated.

Virtanen et al. [29] and Vives et al. [32] found that fixed-term employees are characterized by a higher level of hostility, aggravation, and depression. The research conducted for the purposes of this article revealed only one person who reported deteriorated health manifested by temper tantrums and another by depression.

The research also revealed a relationship between the length of service and the perceived impact of non-standard forms of employment on mental health. People with longer work experience under their current contract were more likely to notice the impact of their form of employment on their mental health. The initial assumption that a worker employed on a non-standard form of employment but working for a given employer for a relatively long time should experience less job insecurity—and thus less of a negative impact from the form of employment on their mental health—was not confirmed. In previous studies, a negative impact on mental health from longer work experience in non-standard forms was demonstrated in relation to temporary workers [72]. It is worth emphasizing that in the present research project, the respondents connected the symptoms of deteriorated mental health with their personality traits and the nature of their work. Healthcare workers indicated an emotional connection with their work, while others complained about the inappropriate behavior of their clients, for example. This highlights the number of factors that can influence one’s perceived health status.

Finally—as the research shows—one important variable that determines the impact of non-standard employment on workers’ health is their reason for taking up employment. Being unable to choose the form of employment is related to a perceived negative impact of the form of employment on physical health rather than mental health. However, it should be stressed once again that the respondents covered by the research were clearly referring to the nature of the job itself (e.g., carrying heavy objects). Perhaps, having no alternative, people accept hard work, which comes with poor working conditions and physical ailments.

## 5. Conclusions

It has been emphasized in the article that many projects have so far used the vague category of precarious employment. The need to take into account the differences between non-standard and precarious employment was also highlighted. Since the researcher’s task is to precisely define the subject of research, and to account for national differences, this article analyzed two specific forms of non-standard employment, i.e., self-employment and mandate contract, as well as two dimensions of health (physical and mental health).

As the research shows, the majority of people employed in non-standard forms of employment believe that this choice does not factor into their health. Moreover, in the process of statistical analysis, a correlation was demonstrated between the perceived impact of a given form of employment on physical and mental health and such variables as the length of service and the reason for taking up employment. These findings constitute a contribution to the development of knowledge regarding the impact of non-standard forms of employment on workers’ health.

The fact that, in the respondents’ perception, their form of employment does not affect their health, in contrast to the results of previous studies indicating a negative health impact from precarious forms, highlights the importance of the proposal to research the category of non-standard forms of employment separately and to not automatically extend conclusions on precarious employment to the whole category of non-standard forms of employment. The differences presented herein regarding the perceived impact of mandate and self-employment contracts on health support the proposal to consider the nature of individual forms of non-standard employment in research. In turn, the failure to demonstrate an impact from variables such as the respondents’ gender and age, yet revealing the importance of the length of service, confirms the need to design research that would address the impact of non-standard forms of employment on workers’ health while taking into account new variables.

When identifying directions for further research, it is worth first of all eliminating the limitations resulting from this study. The main limitation is the size of the research sample, the distribution of certain characteristics of which made it impossible to analyze more deeply. For example, Hammarström et al. [73], when conducting their study in Sweden, found that fixed-term employees—but only those with a low level of education—were characterized by more symptoms of malaise and worse health than permanent employees with a similar education. Unfortunately, the demographics of the research sample used in the present study did not allow for analysis according to the respondents’ level of education.

This research was carried out during the Covid-19 pandemic (July–September 2020). The global health crisis may have affected the reliability of the responses, since it has been empirically proven that overall job security and self-rated health decreased during this period [74,75]. Mental health and well-being were affected the most in the initial phase of the Covid-19 pandemic [76] because of the dynamic changes in the way of working and living such as, e.g., a switch to remote working, the reduction of interactions with other people, and the fear of infection [77,78,79]. Therefore, it is worth conducting similar research after the Covid-19 pandemic and comparing the results.

Although many variables related to non-standard employment’s effect on health status were analyzed in this research, the collected responses highlighted the characteristics of the work itself as well as the personal traits of the respondents. In turn, referring to the need to distinguish non-standard forms of employment from precarious ones, it is worth conducting an in-depth analysis to address the provisions of contracts between workers and companies in future research projects. It may turn out that the non-standard form of employment is much more beneficial for a worker than the standard one. Favorable provisions may—as in the studies presented here—refer to the length of the notice period, the level of remuneration, severance pay in lieu of a lengthy notice period, etc. This illustrates the importance of taking into account additional variables in order to investigate the impact of work based on non-standard forms of employment on workers’ health and conducting more comprehensive research, including some that combine a quantitative approach (survey) with a qualitative one (in-depth interviews).

Finally, although the traditional labor contract is still the prevailing form of employment in EU countries, European labor markets are characterized by increasingly diverse forms of employment. Moreover, new, flexible forms of employment are expected to continue to grow due to the requirements of the Fourth Industrial Revolution and the circular economy [1]. This entails continuing research on the impact of the changing forms of employment on workers’ health. They can also address the topic from an economic perspective, covering the relationship between the financial benefits gained from non-standard employment and the costs spent on health care (at the individual, employer, and national level).

## Figures and Tables

**Table 1:** Number of people employed in Poland based on non-standard forms of employment based on [63].

Form of Employment	Number of Employees (in Thous.)	
	Reference Year: 2017	Reference Year: 2018
Self-employed persons	1200	1300
Persons working based on the mandate contract and who are not employed based on employment contract (from 1 January to 31 December)	986.6	998.9
Persons working based on the contract to perform a specified task and who are not employed based on employment contract (from 1 January to 31 December)	121.2	105.7

**Table 2:** Characteristics of the research sample.

Criterion	Item	Number of Respondents (*n* = 200)	% in the Research Sample Population
Form of employment	mandate contract	100	50
	self-employment	100	50
Gender	female	103	51.5
	male	97	48.5
Age (in years)	under 30	31	15.5
	30+ but less than 40	74	37.0
	40+ but less than 50	70	35.0
	50 and more	25	12.5
Reason for taking up work based on non-standard form of employment	lack of other possibilities	94	47.0
	financial benefits	95	47.5
	as a trial	11	5.5
Length of service in the current non-standard form (years)	1+ but less than 3	93	46.5
	3+ but less than 5	57	28.5
	5+	50	25.0

**Table 3:** The frequency of responses regarding the impact of the form of employment on workers’ physical and mental health.

			In Your Opinion, Does the Form of Contract (Civil-Law Contract) Have an Impact on Your Physical Health?		Total	In Your Opinion, Does the Form of the Contract (Civil-Law Contract) Have an Impact on Your Mental Health?		Total
			Yes, It Has a Negative Impact	It Has No Impact		Yes, It Has a Negative Impact	It Has No Impact	
Form of employment	Mandate contract	Count	29	71	100	8	90	98
		%	29.0%	71.0%	1000%	8.2%	91.8%	100.0%
	B2B contract	Count	10	84	94	18	68	86
		%	10.6%	89.4%	1000%	20.9%	79.1%	100.0%
Total		Count	39	155	194	26	158	184
		%	20.1%	79.9%	1000%	14.1%	85.9%	100.0%

**Table 4:** The frequency of responses regarding the impact of the form of employment on workers’ mental health based on the criterion of the length of service.

			Does the Form of Contract (Civil Law Contract) Have an Impact on Your Mental Health?		Total
			Yes, It Has a Negative Impact	It Has No Impact	
Period of employment for the current company based on the present form of employment	1–3 years	Count	6	84	90
		%	6.7%	93.3%	100.0%
	3–5 years	Count	5	44	49
		%	10.2%	89.8%	100.0%
	More than 5 years	Count	15	30	45
		%	33.3%	66.7%	100.0%
Total		Count	26	158	184
		%	14.1%	85.9%	100.0%

**Table 5:** The frequency of responses regarding the impact of the form of employment on workers’ physical health based on the reason for taking up employment.

			In Your Opinion, Does the Form of the Contract (Civil-Law Contract) Have an Impact on Your Physical Health?		Total
			Yes, It Has a Negative Impact	It Has No Impact	
The reason for taking up employment based on a civil-law contract	No other options	Count	29	64	93
		%	31.2%	68.8%	100.0%
	Financial benefits	Count	10	80	90
		%	11.1%	88.9%	100.0%
	As a trial	Count	0	11	11
		%	0.0%	100.0%	100.0%
Total		Count	39	155	194
		%	20.1%	79.9%	100.0%

## Data Availability

The data presented in this study are available on request from the corresponding author.
